# The Story of the Insane from Year to Year

**Published:** 1894-07-21

**Authors:** 


### THE STORY OF THE INSANE FROM YEAR TO YEAR.

(SEVENTH SERIES.)

### CITY OF LONDON ASYLUM.

This asylum is situated near Stone, Dartford, Kent, and it now issues its twenty-eighth annual report, from which we learn that the numbers on January 1st, 1893, were 427, and on December 31st they had risen to 446. Exactly 100 patients were admitted in the twelve months; 42 recovered and 26 died. Twenty-two were driven mad by drink, and hereditary tendency accounts for 25. Only three of the deaths were caused by brain disease, and the same number by phthisis. The recoveries work out at 53·16 per cent., and the deaths at 8·49, the former being much above the average, and the latter somewhat under it. Although the death-rate was low, Dr. White had a varied experience of disease, and his *clientele* included typhoid fever, influenza, scarlet fever, and epidemic diarrhœa. Some of these were due to defective sanitary arrangements, since remedied. Dr. White attributes his high recovery rate among the males, to the active outdoor employment which the farm affords, and there can be no doubt of the immense value of steady employment in the treatment of the insane. During the year five nurses and one attendant passed the examination for the nursing certificate of the Medico-Psychological Association, and Dr. White thinks this training of the staff is not an unmixed blessing, as three of his charge nurses left to obtain employment as private nurses. No doubt this is a contingency which everyone has to think about, as some nurses will be tempted away by higher pay and easier work; but they will not be the best of the staff, for a reflecting man or woman will weigh the regular salary; the fixed hours and the prospective pension against the inducements of private nursing, and most likely decide to stay where he or she is. The committee say they "have every reason to be gratified at the efficient manner in which the officers and servants continue to discharge their duties." On looking at the list of officers and salaries, we notice that the assistant medical officer receives only £120 a year, the head attendant £50, and the head nurse £40. All these occupy responsible posts, and must give up the whole of their time to their work; while the chaplain, whose duties must necessarily be of the lightest possible description, receives £200; and the clerk to the visitors, whose duties are lighter still, receives £150. Surely the salaries need revising.

### BELFAST DISTRICT ASYLUM.

The accommodation is stated to be for 550 patients, but, as seems usual in Irish asylums at present, the numbers resident are far in excess of the accommodation, being 691 on the first day of the year, and 706 on the last day of December. The admissions were 210, the recoveries 92, and the deaths 45. The recoveries stand at 43·3 of the admissions, and the deaths at 6·4 on the average number resident. Table XI. tells us that 81 of the patients resident cannot read nor write, and the same number can read only. Table XIII. shows that 172 belong to the Church of Ireland, 284 are Presbyterians, 221 are Catholics, and 29 are of "other denominations." In next year's statistics we should much like to see a third table compiled showing the relations of the various religions to education. The Commissioner says he "found all parts of the asylum clean, and bearing in mind the great overcrowding which exists, its administration must be considered to be in many respects satisfactory." But further on he comments on the dreary character of the wards at the rear of the asylum, to the insufficiency of chapel, laundry, stores, workshops, &c., and gives us the agreeable information that a site for a new asylum is to be acquired. We would, however, ask with all deference who is responsible for the delay, and why is an asylum allowed to accumulate 150 patients in excess of its proper number, and no steps taken to remedy the evil? The cost per head per annum was £21 5s. 8d.

### CORK DISTRICT ASYLUM.

Here the accommodation is considerably in excess of the numbers resident. The former is for 1,344 patients, and the numbers on January 1st, 1893, were 558 men and 534 women, a total of 1,092, which had increased to 1,123 by the end of December. The admissions were 260, the recoveries 88, and the deaths 105. The recoveries stand at 33·8 per cent. on the admissions, and the deaths at 9·4 on the average resident. Both of these percentages are a little under the average. Drink is given as the assigned cause in 32 cases, and hereditary influence in 17 only; but Dr. Woods states, and correctly so, that "intemperance is a far more frequent cause of insanity than is assigned, and hereditary predisposition is always reluctantly acknowledged by the friends, and should hold a much more prominent position as a cause of insanity than it does in these returns," and further on, with more truth than originality, he records his opinion that the "free discharge of weak-minded but harmless patients, and the too early removal of those recovering, has tended to increase considerably the number of insane who break down from heredity." Medical superintendents are fond of pointing to a high recovery rate; but if they would reflect on the fact that the higher the recovery rate in this generation the greater will be the number of insane people in the next, they might perhaps be induced to hide the results of their treatment as one of those things better not enlarged on. The rate per head per annum is £20 12s.

